# Effects of Hippotherapy and Horse-Riding Simulators on Gross Motor Function in Children with Cerebral Palsy: A Systematic Review

**DOI:** 10.3390/jcm14010283

**Published:** 2025-01-06

**Authors:** Antonio Ortega-Cruz, Víctor Sánchez-Silverio, Víctor Riquelme-Aguado, Jose Luis Alonso-Perez, Vanesa Abuín-Porras, Jorge Hugo Villafañe

**Affiliations:** 1 Department of Physiotherapy, Faculty of Medicine, Health and Sports, Universidad Europea de Madrid, 28670 Villaviciosa de Odón, Spain; antonio.ortega@universidadeuropea.es (A.O.-C.); joseluis.alonso@universidadeuropea.es (J.L.A.-P.); abuinvanesa@gmail.com (V.A.-P.); mail@villafane.it (J.H.V.); 2 School of Applied Health Sciences, Pontificia Universidad Católica Madre y Maestra, Autopista Duarte Km 1 1/2, Santiago De Los Caballeros 51000, Dominican Republic; ve.sanchez@ce.pucmm.edu.do; 3 Department of Basic Health Sciences, Rey Juan Carlos University, 28933 Madrid, Spain; 4 Grupo de Investigación Consolidado de Bases Anatómicas, Moleculares y del Desarrollo Humano de la Universidad Rey Juan Carlos (GAMDES), 28922 Alcorcón, Spain; 5 Musculoskeletal Pain and Motor Control Research Group, Faculty of Sport Sciences, Universidad Europea de Madrid, 28670 Villaviciosa de Odón, Spain

**Keywords:** hippotherapy, horse-riding simulators, cerebral palsy, gross motor function, gross motor function measure

## Abstract

**Background/Objectives**: Cerebral palsy (CP) can have a negative impact on gross motor function. Conventional hippotherapy and horse-riding simulators (HRS) have shown promising results on gross motor function in populations with neurological disorders. This review aims to update the knowledge on the effectiveness of hippotherapy on gross motor function in children with CP. **Methods**: A search was conducted in Academic Search Ultimate, CINAHL, Medline complete, and PEDro covering publications between 2012 and 2022. Two authors identified studies that met the inclusion criteria; a third author resolved discrepancies. Studies were included if they analyzed the effects of hippotherapy on the gross motor function of children with CP. The quality of the methodology was assessed according to the PEDro scale. **Results**: Of the 150 studies initially identified, 9 were included in this review. The studies showed fair (N = 3) and good (N = 6) methodological quality on the PEDro scale. The majority used conventional hippotherapy (N = 7), while a minority used HRS (N = 2). The most commonly used protocol for conventional hippotherapy was 1–2 sessions of 30–45 min per week for 8 weeks (N = 4), whereas for HRS, these protocols were varied. Seven studies on conventional hippotherapy and one study on HRS showed improvements in gross motor function. However, the hippotherapy protocols were not very standardized and the samples were neither homogeneous nor representative. **Conclusions**: Conventional hippotherapy and HRS appear to have evidence to support their benefits on gross motor function in children with CP. However, more clinical trials with standardized protocols and more representative samples are needed to confirm these effects.

## 1. Introduction

Cerebral palsy (CP) is the most common cause of physical disability in children [1]. Studies have estimated its prevalence at approximately 2.11 cases per 1000 births in developed countries since 1985 [2]. Other authors suggest that these figures may range from 1.5 to 3 when considering high-, middle-, and low-income countries [3]. It should be noted that in 90% of cases, MRI reflects findings such as brain malformations, in utero stroke, or white matter loss [4,5,6].

Children with CP often have impairments related to postural control, gait, and gross motor function, as well as sensory impairment, spasticity, and visual and intellectual impairment [2]. Gross motor function involves the large-muscle actions that allow for movement of the whole body or large segments of the body. These functions include locomotor skills (e.g., running, jumping, or sliding), static balance (e.g., standing/sitting), dynamic balance (e.g., walking), and object control/handling skills (e.g., throwing, catching, or hitting objects) [7,8]. This function is used to describe children’s ability to walk and perform activities of daily living. Most children with CP have gross motor impairments that interfere with the execution of patterns such as walking and require the use of external aids, such as walkers or wheelchairs, for daily functioning [9].

Hippotherapy is defined as equine-assisted therapy that uses movements characteristic of horses during a rehabilitation process [10]. Its effects on CP have been proposed that the child receives impulses from the horse that stimulate the activation of his sensory, neuromotor and cognitive systems [11]. This has led to this approach being labeled as a therapeutic alternative to be administered to children with CP. However, conventional hippotherapy may have limitations related to distance, time and cost of its application; in these cases, horse-riding simulators (HRS) are often used. Researchers have defined HRS as a robotic device designed to replicate the therapeutic benefits of horseback riding. It consists of a dynamic saddle mechanism that produces three-dimensional movements closely mimicking the natural gait pattern of a horse. These movements are intended to stimulate the rider’s neuromuscular responses, promoting balance, coordination, and core strength. By simulating the horse’s rhythmical motion, HRS provides a controlled, safe, and repeatable environment for therapeutic interventions, often used in rehabilitation programs for individuals with physical, neurological, or developmental disorders [12].

A review of children with CP concluded that conventional hippotherapy may be effective in improving balance and reducing spasticity. The authors noted that hippotherapy has been suggested as an approach that may have positive effects on gross motor and hand function in children with CP [13]. Their impairment in CP would not only affect general body movements, but also specific fine motor patterns that may be essential for the child’s psychomotor development. In this context, the promotion of hippotherapy, in its conventional form or through HRS, could play an essential role in the recovery of motor functions in children with CP. Therefore, the aim of this review is to update the knowledge on the effectiveness of hippotherapy on gross motor function in children with CP.

## 2. Materials and Methods

### 2.1. Search Strategy

Present systematic review was performed according to the Preferred Reporting Items for Systematic Reviews and Meta-Analyses (PRISMA) guidelines. The literature search identified studies that examined the effect of hippotherapy on gross motor function in children with CP. The search was conducted in the following scientific sources: Academic Search Ultimate, CINAHL, Medline complete, and PEDro. The following MESH terms were used in these databases: “Hippotherapy” OR “Horse therapy” OR “Equine therapy” OR “Equine assisted therapy” OR “Riding therapy” OR “Horse riding simulator” AND “Children cerebral palsy” AND “Gross motor function classification” OR “Gross motor function measure”. Two authors searched for studies and assessed potentially eligible studies based on title and abstract. A third author was responsible for resolving discrepancies in this selection process. Each author independently reviewed the abstract and full text of the article according to the inclusion and exclusion criteria defined in this review.

### 2.2. Selection of Studies

#### 2.2.1. Types of Studies

To be eligible for inclusion in this qualitative synthesis, studies had to be clinical trials published between 2012 and 31 August 2022. Studies were included if they scored ≥5 on the PEDro scale and evaluated the effects of hippotherapy and HRS on gross motor function. In addition, studies had to compare two different interventions or the same intervention (hippotherapy or HRS) with different durations of application or in different subjects according to the Gross Motor Function Classification System (GMFCS). Studies that were not published in English were excluded. Systematic reviews, meta-analyses, case reports, letters to the editor, and congress communications were also excluded.

#### 2.2.2. Type of Participants

Participants had to be children of either sex diagnosed with CP. Studies which included children or adults with disabilities other than CP were excluded.

#### 2.2.3. Data Extraction

All relevant studies from the data sets were analyzed by two reviewers who also performed the extraction independently. A third author resolved discrepancies during this process. The information extracted from each study included the authors’ names, year of publication, country where the study was conducted, purpose of the study, study design, characteristics of the population (Number, gender and age of participants), clinical outcome measures, treatment, and reported results. This format was adapted from the Cochrane Handbook for Systematic Reviews of Interventions—version 5.1.0. Data on type of CP and level of disability according to GMFCS were also extracted from the studies.

### 2.3. Quality of Studies

Methodological quality was assessed using the PEDro scale. This scale consists of 11 items that assess the following criteria: (1) eligibility criteria, (2) random allocation, (3) concealed assignment, (4) similarity at baseline, (5) subject blinding, (6) therapist blinding, (7) assessor blinding, (8) >85% follow up for at least one key outcome, (9) intention-to treat analysis, (10) between-group statistical comparison for at least one key outcome, and (11) point and variability measures for at least one key outcome. With the exception of the first item, all other criteria are scored as present (1) or absent (0). The sum gives a score between 0 and 10. In this score range, the PEDro scale suggests the following cut-off points: excellent quality (9–10 points), good quality (6–8 points), fair quality (4–5 points), and poor quality (<4 points) [14]. Two authors evaluated the clinical trials, with a third author making the final decision in case of disagreement.

## 3. Results

### 3.1. Selection of Studies

**Figure 1:**
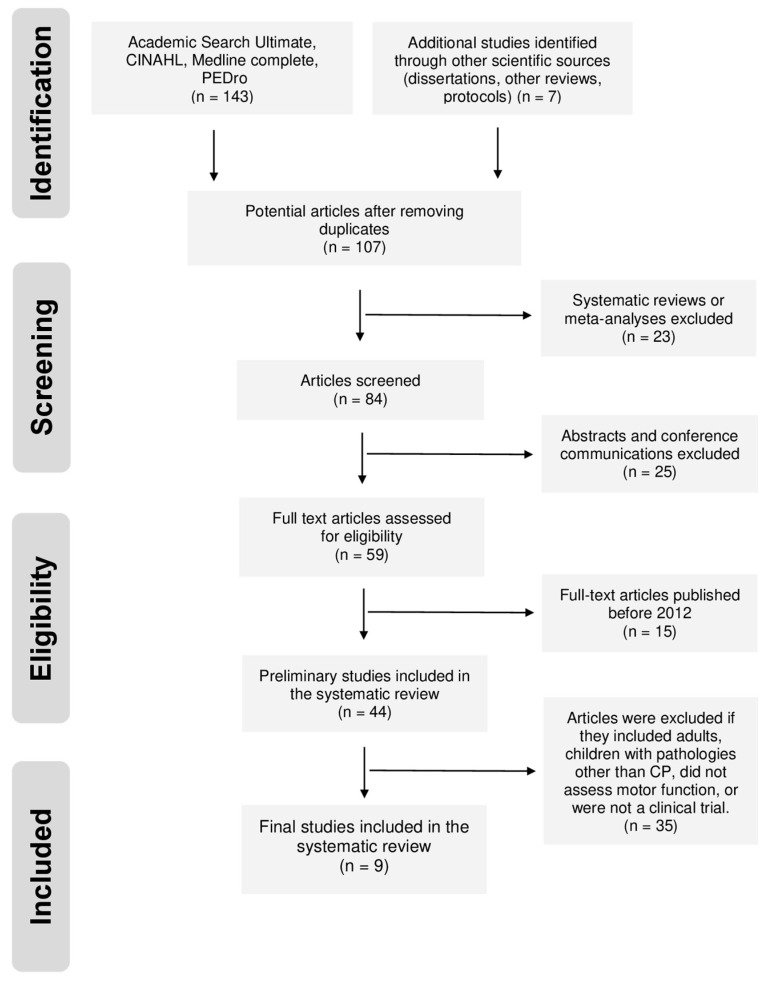
Flow chart diagram illustrating the database searches, number of publications identified and screened, and final full texts included in the systematic review.

A total of 150 studies were identified and 107 potential articles were selected after eliminating duplicates. By continuing the screening process through titles and abstracts as well as inclusion criteria, 44 studies were considered eligible for the qualitative synthesis of this review. Of these studies, 35 were excluded because they included as a population adult patients or children with pathological conditions other than CP. Other studies were excluded because they did not assess gross motor function or were not considered clinical trials. Finally, 9 studies were analyzed in this systematic review (Figure 1).

### 3.2. Characteristics of the Studies

Table 1 shows the characteristics of the studies and their respective participants. The publications ranged from August 2012 to March 2021. Five studies were developed in different countries [15,16,17,18,19], while four studies were conducted in South Korea [20,21,22,23]. The smallest and largest numbers of children evaluated were 20 and 91, respectively, with an age range from 2 to 18 years. In addition, the smallest number of female participants was 8 and the largest was 42, while the smallest and largest numbers of male participants ranged from 11 to 49.

Seven of the studies specified the type of CP [15,17,18,20,21,22,23], while only two did not specify this information. Of the studies that reported the type of CP, all included children with spastic CP; in this group, only one study also included dyskinetic and ataxic CP. Furthermore, among these studies, diplegia was present in six studies [15,17,18,21,22,23], whereas hemiplegia was present in three studies [17,21,23] and quadriplegia in only one study [22]. For GMFCS levels, only one study did not define the functional level of its population [23]. Of the remaining studies, two included children with GMFCS functional levels up to V [16,19], three included a population with functional levels up to IV [20,21,22], one included a population with functional levels up to III [19,20], and one included a population with functional levels up to II [17].

### 3.3. Quality of Studies

The results regarding methodological quality are shown in Table 2. Below the cut-off points, three studies were of fair quality [21,22,23] and six of good quality [15,16,17,18,19,20]. Five studies explained their sample calculation [18,19,20,21,22], while four did not provide this information [15,16,17,23]. Only one study did not use randomization methods for its participants [22]. However, of the eight studies that used randomization, only three implemented blinding in the allocation of sites between groups [15,16,19]. Of the blinding strategies used in the methodology, three studies used blinding of participants [18,19,20,22], five blinded assessors [17,18,19,20,22], and only one blinded the clinicians delivering the intervention [20].

### 3.4. Data from Studies

**Figure 2:**
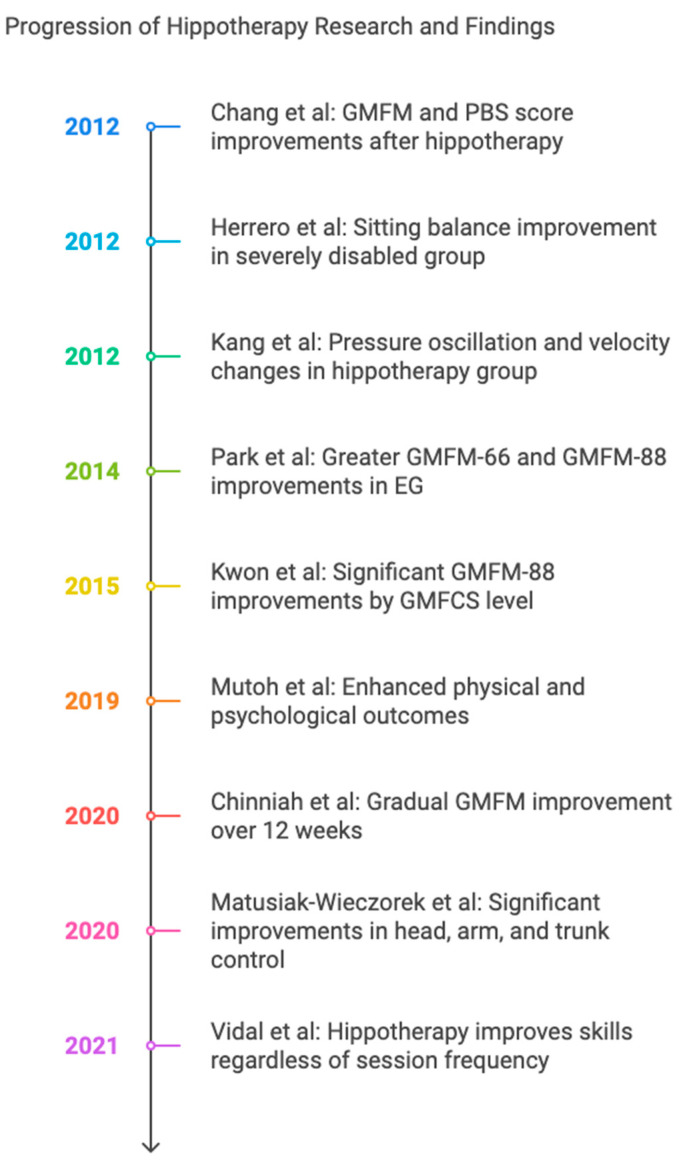
Visual representation abstract of results [15,16,17,18,19,20,21,22,23].

The information on the methodology applied to the participants is presented in Table 3. A Visual representation abstract of results is presented in Figure 2. 

#### 3.4.1. Measurement of Variables

Eight of the nine studies used the Gross Motor Function Measure (GMFM) to assess gross motor function. Of these studies, two used the GMFM-88 [15,22] and three used the GMFM-66 [16,18,19], while two studies used both versions [20,21]. Furthermore, one of the studies used the Sitting Assessment Scale (SAS) to assess upper limb motor function [17]. Balance was another function assessed using the Pediatric Balance Scale (PBS) [20,22], the SAS [16,17], dimension B of the GMFM [16], and a strength platform [23]. In addition, some studies assessed functionality using the Pediatric Evaluation of Disability Inventory (PEDI) [19,21] and the 5 m walk test [18].

#### 3.4.2. Characteristics of the Interventions

To evaluate the effects of hippotherapy on gross motor function, seven studies used two analysis groups [15,16,17,19,21,22] and two studies used three groups [17,23]. The experimental group underwent hippotherapy delivered conventionally [17,18,19,20,21,22,23] or using an HRS [15,16]. Conventional hippotherapy consisted of the child sitting on the horse while the horse moved in different rhythms or changed patterns or directions. In sessions with the HRS, the child sat on a simulator covered with wool while anterior, lateral, and posterior movements were performed. On the other hand, the comparative/control group protocol focused on the variation of some process of hippotherapy used in the experimental group, the performance of conventional physiotherapy, or the non-performance of an intervention.

In the studies that used conventional hippotherapy, four applied between one and two sessions of 30–45 min/week for a period of 8 weeks [18,21,22,23], while others implemented this protocol but for periods of 12 [17], 16 [19], and 48 weeks [18]. For HRS, one to three sessions of 15 min/week were used for 10 [16] and 12 weeks [15]. Importantly, eight studies included therapists trained to deliver the hippotherapy sessions [15,17,18,19,20,21,22,23]. Of these, five reported that the therapists were certified in hippotherapy [18,19,20,21,22].

#### 3.4.3. Effects of Hippotherapy

All studies reported positive effects of hippotherapy on one or more of the variables analyzed.

In one study, gross motor function improved in two groups with different levels of disability (GMFCS I–II vs. GMFCS III–IV) [22]. On the other hand, a study that analyzed the frequency of sessions per week (one session vs. two sessions) found positive effects on the GMFM-66 in both groups before and after the assessment, but no differences when comparing these groups [19]. However, another similar study found significant improvements in motor function measured by the SAS in the group that received two sessions/week [17]. In other studies that strictly considered both an experimental and control group, significant improvements in the GMFM-66 and GMFM-88 were found only in the experimental group at the end of the intervention [18,20], while in two other studies, these significant improvements were reflected in both groups [15,18,20,21]. However, when establishing a comparative framework, motor function benefits were significant in the experimental group compared to the control group in all four studies [15,18,20,21]. It should be noted that only one study found no improvement in gross motor function when comparing the experimental group to controls [16].

Regarding balance, one study showed improvements in PBS in groups with different levels of disability [22]. Other studies showed these improvements by the SAS in children who underwent a greater number of sessions of conventional hippotherapy per week [17], and by dimension B of the GMFM in children who underwent HRS [16]. Similarly, in two studies, the experimental group showed significant improvements in balance at the end of conventional hippotherapy compared to controls, as assessed by the force plate [23] and the PBS [20]. Of the studies that assessed functional performance, all showed better performance in the experimental group on the PEDI-FSS [21], the 5 m walk test [18], and the PEDI.

## 4. Discussion

Over time, hippotherapy has evolved from an unconventional method to a useful approach in clinical practice. The purpose of this qualitative synthesis was to update knowledge regarding the effects of hippotherapy on gross motor function in children with CP. Regardless of etiology, CP is a condition that can affect gross motor function and performance in daily living [24]. Hippotherapy has been proposed as a method that can have positive effects on motor, psychological, cognitive, and social aspects in children with CP [25]. The present review analyzed 9 studies related to hippotherapy in CP.

The vast majority of studies showed positive effects on gross motor function in children with CP. Some authors state that hippotherapy may be beneficial for motor function in CP because it requires the user to perform three-dimensional movements that stimulate balance-related systems [11]. These balance functions are often necessary to perform efficient gross movements with the limbs. In the studies reviewed, these balance demands were required by movements generated by conventional hippotherapy [17,18,19,20,21,22] or HRS [16,17]. Notably, in six of the seven studies using conventional hippotherapy, the protocol was supplemented with activities involving head, trunk, and limb movements [17,18,19,20,22,23,24]. This is consistent with a review that confirmed, through an analysis of the evidence on hippotherapy between 1980 and 2018, that 100% of the studies used functional activities in their respective neurological populations. It should be added that, in this review, 51% of the studies were conducted in children with CP [26].

With the exception of two studies [16,19], the sample in the remaining studies showed a greater predominance of one type of CP. All of the studies included children with spastic CP [15,17,18,20,21,22,23], whereas only one study also included children with other types of CP [20]. This is consistent with research suggesting that spastic CP tends to be the most prevalent in adults and children with motor disorders [27,28]. Other authors suggest that selective motor control and spasticity in this type of CP may be influential factors in gross motor function [29] A recent meta-analysis even found that conventional hippotherapy and HRS may have beneficial effects on spasticity in children with CP, especially in the lower extremities [30]. Considering the above findings, it may be understandable that most of the reviewed studies showed positive effects of hippotherapy on the gross motor function of the children evaluated.

On the other hand, the timing of the protocol in the studies reflected certain similarities. In the vast majority, one to two conventional hippotherapy sessions of 30–45 min/week were applied for 8 weeks [20,22,23,24], although in the HRS studies, these sessions were applied for 15 min over a range of 10 to 12 weeks [15,16]. In the cited review, which analyzed studies between 1980 and 2018, conventional hippotherapy protocols generally consisted of one to two sessions/week [26]. Although not a conclusive finding, these consistencies could, tentatively, serve as a basis for making decisions about the frequency of weekly sessions that should be considered in a hippotherapy protocol. On another note, in the present review, the measures used to assess gross motor function showed greater consistency. Seven studies used the GMFM [15,16,18,19,20,21,22], while only one study chose part of the SAS to assess these functions in the population studied [17]. This finding may be understandable as the GMFM is one of the main measures used to assess gross motor function in children with CP.

It should be added that, although eight studies included professionals in the application of the protocols, only five stated that they were carried out by professionals certified in hippotherapy [14,17,18,19,22]. In a review that analyzed 78 studies, 78% stated that hippotherapy was applied by health professionals, 71% of whom were physiotherapists [26]. Although hippotherapy suggests the involvement of a rehabilitation professional, its proper implementation may depend on including certified individuals who can extrapolate its theoretical underpinnings to a more pragmatic setting. Future research should consider the formation of multidisciplinary teams to delve deeper into the psychological and cognitive aspects of these patients

In most of the studies in this review, hippotherapy protocols produced benefits in gross motor function. One of the studies confirmed improvements in two groups with different degrees of disability, although these effects were slightly greater in those with more severe disability [22]. Another study comparing the weekly application of hippotherapy observed improvements in gross motor function regardless of whether it was applied in one or two sessions per week [19]. However, a study with a similar aim showed more significant effects in children who received two sessions per week [17]. These differences could be explained by processes related to motor learning, since there is evidence that a greater number of practical sessions may be associated with better acquisition of the motor skills trained. Moreover, in the first study, these sessions were applied for 12 weeks [19], while in the second study, the protocol was extended to 16 weeks [17].

On the other hand, all studies that compared an experimental group with controls found benefits in gross motor function in the group that received hippotherapy [15,18,20,21]. Three of these studies used conventional hippotherapy [18,20,21], while the remaining study used an HRS [15]. These improvements were consistent with the benefits on gross motor function found in a systematic review that evaluated the effectiveness of conventional hippotherapy with studies up to 2018. However, it should be noted that, similarly to the present study, the protocols of the studies in this review were very heterogeneous [10].

It is important to note that only one of the studies analyzed that used an HRS did not find significant effects on gross motor function compared to controls. However, it did show improvements in balance assessment [16]. These improvements were mirrored in other studies that assessed this skill in parallel with conventional hippotherapy [17,20,22,23]. These findings are consistent with a meta-analysis that demonstrated the benefits of hippotherapy on balance in pediatric and adult neurological patients [31]. Balance plays an essential role in the functionality of a child with CP, as good stability in the axial musculature can facilitate more efficient gross movements in the extremities. As the authors state, the demands derived from the three-dimensional movement of hippotherapy require the child to have postural stability that can improve static or dynamic balance [32].

As can be seen, most studies have shown positive effects of hippotherapy on gross motor function in children with CP. However, it is necessary to point out that the studies included in the review have a number of limitations that need to be exposed. First, only two of the nine studies used HRS in children with CP. This creates the need for more evidence with HRS to confirm its effects on gross motor function. Second, the population studied was very heterogeneous in its level of disability within the GMFCS. Although one of the studies reported similar improvements in children with different levels of disability [22], the results of this study point to the importance of validating these findings using a more standardized hippotherapy protocol. When interpreting the results of this review, the aspect of heterogeneity in the populations must be considered when extrapolating them to a general population of children with CP. Third, seven of nine studies analyzed children diagnosed with spastic CP only [15,16,17,18,21,23], which may make it difficult to generalize the results to other types of CP, such as athetotic or ataxic CP. In addition, seven out of nine studies suggested that a larger number of participants was needed to validate their results [15,16,17,18,19,21,22]. It is appropriate to have more evidence to evaluate the effects of hippotherapy not only in different types of CP, but also in more representative samples. Fourth, although the hippotherapy protocol showed certain consistencies in the time of application, the tasks implemented in it were heterogeneous, especially in the studies that used conventional hippotherapy.

On the other hand, the lack of long-term assessment in the studies increases the need to develop follow-up studies to confirm whether the effects of hippotherapy on gross motor function can be sustained over time. Finally, although the studies in this review were required to have a score ≥5 on the PEDro scale, clinical trials with more stringent blinding techniques are needed, as few studies blinded participants [18,20,22] and clinicians applying the hippotherapy protocol [15].

Despite the limitations mentioned above, the present review shows a trend over the last 10 years that is relatively consistent with the benefits shown by the evidence. Hippotherapy is gaining a more prominent role in the field of rehabilitation. As a technique, it has demonstrated positive changes when used in conjunction with other interventions or when integrated as an adjunct to achieve a specific treatment goal. For this reason, in addition to the application of a protocol, it is essential to promote the training of professionals according to the guidelines of this therapeutic approach. Furthermore, the results related to HRS open a window into its therapeutic implications on the clinical side, since this alternative could be considered in clinical settings that may not have the possibility to apply traditional hippotherapy. Finally, a consistent point in the studies was the use of the GMFM to assess gross motor function. This may provide a framework to guide practitioners in the selection of tools that can reliably assess these functions in children with CP.

### Applications for Clinical Practice and Future Research Lines

Based on the findings from the present systematic review, both conventional hippotherapy and HRS have shown significant benefits in improving gross motor function in children with CP. For optimal clinical application, these interventions should be administered by certified therapists, preferably within multidisciplinary teams to address not only motor development, but also psychological and cognitive aspects of the patients. Research suggests that the duration of each session should range from 30 to 45 min, with a frequency of one to two sessions per week. The most effective intervention periods observed in the studies ranged from 8 to 12 weeks, ensuring sufficient time for measurable improvements. From an economic perspective, hippotherapy with simulators could be considered a more affordable option for some clinical settings, making the therapy more accessible while still yielding positive outcomes. It is important to note that these recommendations are based on the general findings from the literature, and clinicians should tailor their approach based on the individual needs of their patients, considering factors such as the level of impairment and available resources. Additionally, future studies should consider long-term follow-ups to evaluate the sustainability of these benefits and further refine the guidelines for the application of both therapies. Future clinical trials could focus on further analyzing this aspect by conducting similar interventions in patient groups stratified according to different GMFCS levels. This approach would allow for a more precise evaluation of specific responses and the optimization of therapeutic strategies tailored to each level of impairment.

## 5. Conclusions

Based on the literature in this review, conventional hippotherapy and HRS appear to have evidence to support their benefits regarding gross motor function in children with CP. Similarly, it has been suggested that this approach may not only help to improve gross motor function, but may also have an impact on balance in children with CP. However, further clinical trials with more standardized hippotherapy protocols and more homogeneous and representative samples are needed. In this regard, it is advisable that these trials include follow-up assessments to confirm the effects of hippotherapy in the long term. Finally, more studies are needed to validate the effects of HRS on gross motor function in children with CP.

## Figures and Tables

**Table 1:** Characteristics of the studies and the population. CP: Cerebral palsy; GMFCS: Gross Motor Function Classification System; RCT: Randomized Controlled Trial; n: number of children; M: male; F: female.

Author and Year	Location of Study	Aim of the Study	Study Design	Population
Chang et al., 2012 [22]	South Korea	To investigate whether hippotherapy could improve the functional performance of preschool- and school-aged children with spastic bilateral CP	Clinical trial	n: 33 children with CP Type of CP: spastic (quadriplegia and diplegia) Gender: 19 M/14 F Age: 6 years (Mean) GMFCS level: I–IV
Herrero et al., 2012 [16]	Spain	To investigate whether hippotherapy (when applied by a simulator) improves postural control and balance in children with CP	RCT	n: 38 children with CP Type of CP: not specified Gender: 24 M/14 F Age: entre 4–18 years GMFCS level: I–V
Kang et al., 2012 [23]	South Korea	To verify the effect of hippotherapy on the sitting balance of children with severe CP by comparing hippotherapy, physical therapy, and a control	RCT	n: 43 children with CP Type of CP: spastic (hemiplegia and diplegia) Gender: 22 M/21 F Age: 8 years (Mean) GMFCS level: not specified
Park et al., 2014 [21]	South Korea	To investigate the effects of hippotherapy on gross motor function and functional performance in children with spastic CP	RCT	n: 55 children with CP Type of CP: spastic (bilateral -most frequent- and unilateral) Gender: 25 M/30 F Age: between 3–12 years GMFCS level: I–IV
Kwon et al., 2015 [20]	South Korea	To examine whether hippotherapy has a clinically significant effect on gross motor function in children with CP	RCT	n: 91 children with CP Type of CP: spastic (most frequent), dyskinetic and ataxic Gender: 49 M/42 F Age: between 4–10 years GMFCS level: I–IV
Mutoh et al., 2019 [18]	Japan	To determine how hippotherapy affects the gross motor and gait functions in children with CP and how it may also impact the quality of life of patients’ caregivers	RCT	n: 24 children with CP Type of CP: spastic (bilateral -diplegia-) Gender: 11 M/13 F Age: entre 4–14 years GMFCS level: II–III
Chinniah et al., 2020 [15]	India	To investigate the therapeutic effects of horse-riding simulator on sitting motor function in children with spastic diplegia	RCT	n: 30 children with CP Type of CP: spastic (diplegia) Gender: 13 M/17 F Age: between 2–4 years GMFCS level: I–III
Matusiak-Wieczorek et al., 2020 [17]	Poland	To assess the influence of hippotherapy on posture and body function among children with CP	RCT	n: 45 children with CP Type of CP: spastic (diplegia and hemiplegia -most frequent-) Gender: 25 M/20 F Age: between 6–12 years GMFCS level: I–II
Vidal et al., 2021 [19]	Brazil	To verify whether hippotherapy once or twice a week has a different effect on gross motor function and functional performance in children with CP	RCT	n: 20 children with CP Type of CP: not specified Gender: 12 M/8 F Age: between 2–5 years and 11 months GMFCS level: II–V

**Table 2:** Quality assessment of selected studies using PEDro scale. PEDro: Physiotherapy Evidence Database; (1): present; (0): absent; *: criterion excluded from the total score; C1: eligibility criteria; C2: random allocation; C3: concealed allocation; C4: baseline comparability; C5: blind subjects; C6: blind therapists; C7: blind assessors; C8: adequate follow-up; C9: intention-to-treat analysis; C10: between-group comparisons; C11: point estimates and variability.

Study	C1 *	C2	C3	C4	C5	C6	C7	C8	C9	C10	C11	Total
Chang et al., 2012 [22]	1 *	0	0	1	1	0	1	0	0	1	1	5/10
Herrero et al., 2012 [16]	0 *	1	1	1	0	0	0	1	1	1	1	7/10
Kang et al., 2012 [23]	1 *	1	0	1	0	0	0	1	0	1	1	5/10
Park et al., 2014 [21]	1 *	1	0	1	0	0	0	1	0	1	1	5/10
Kwon et al., 2015 [20]	1 *	1	0	1	1	0	1	1	0	1	1	7/10
Mutoh et al., 2019 [18]	1 *	1	0	1	1	0	1	0	1	1	1	7/10
Chinniah et al., 2020 [15]	1 *	1	1	1	0	1	0	1	1	1	1	8/10
Matusiak-Wieczorek et al., 2020 [17]	0 *	1	0	1	0	0	1	1	1	1	1	6/10
Vidal et al., 2021 [19]	1 *	1	1	1	0	0	1	1	0	1	1	7/10

**Table 3:** Outcome measures, intervention characteristics, and results of the studies analyzed. EG: experimental group; CG: control group; n: number of children; CP: cerebral palsy; GMFM-88: Gross Motor Function Measure version 88; GMFM-66: Gross Motor Function Measure version 66; PBS: Pediatric Balance Scale; SAS: Sitting Assessment Scale; PEDI-FSS: Pediatric Evaluation of Disability Inventory-Functional Skills Scale; WHOQOL-BREF: The World Health Organization Quality of Life-BREF.

Study	Outcome Measures	Experimental Group—Control/Comparative Group	Reported Results
Chang et al., 2012 [22]	Gross motor function: GMFM-88 Balance: PBS	Group A (n = 19): GMFCS I–II Group B (n = 14): GMFCS III–IV *Protocol in both groups: hippotherapy (2 sessions of 30 min · week/8 weeks) with riding specialists. The movements of each horse were modified during the treatment sessions according to the child’s needs* (e.g., *walking, changing rhythm or changing patterns and directions*).	Results: the GMFM total score and the scores of dimensions C, D, and E increased after hippotherapy in group B (*p* < 0.05), while only dimension E and the GMFM total score improved in group A (*p* < 0.05). In both groups, the PBS score increased after hippotherapy (*p* < 0.01). Conclusions: hippotherapy can improve gross motor function and balance in pediatric patients with CP without adverse effects. This method is recommended for preschool- and school-aged children with spastic CP.
Herrero et al., 2012 [16]	Gross motor function: GMFM-66. Sitting balance: dimension B of GMFM and SAS	EG (n = 19): consisted of sitting on the hippotherapy simulator with active trunk extension while the simulator was turned on in TRAINING mode (1 session of 15 min × week/10 weeks). CG (n = 19): consisted of sitting on the hippotherapy simulator with active trunk extension while the simulator was turned off (1 session of 15 min × week/10 weeks).	Results: sitting balance (dimension B of the GMFM) improved significantly in the EG, and the effect was greater in the severely disabled group (*p* < 0.05). Improvements in sitting balance were not maintained over the follow-up period. In addition, changes in GMFM and SAS total score were not significant. Conclusions: simulator hippotherapy can improve sitting balance in children with CP who have higher levels of disability.
Kang et al., 2012 [23]	Sitting balance: Force plate	Hippotherapy group (n = 15): received hippotherapy and traditional physiotherapy (stretching program). Hippotherapy consisted of sitting and standing in the saddle, manipulating objects and maintaining posture while the horse moved (2 sessions of 30 min × week/8 weeks). Physiotherapy group (n = 14): received only traditional physiotherapy (2 sessions of 30 min × week/8 weeks). CG (n = 14): did not receive intervention.	Results: center of pressure oscillation and velocity decreased in the hippotherapy group compared to the physiotherapy group and CG (*p* < 0.05). The physiotherapy group showed differences in right/left trajectories and total trajectories as well as right/left velocity and final velocity compared to the CG (*p* < 0.05). Before and after the intervention, improvements in all variables were demonstrated in the hippotherapy group (*p* < 0.05). Conclusions: hippotherapy with traditional physical therapy improves sitting balance in children with severe CP (inability to walk) compared to traditional physical therapy alone.
Park et al., 2014 [21]	Gross motor function: GMFM-66 y GMFM-88. Functional performance: PEDI-FSS	EG (n = 34): hippotherapy was used under the direction of a specialist and a trained assistant (2 sessions of 45 min × week/8 weeks). The child sat on the horse and performed activities that emphasized forward and upward movement to promote active postural control, trunk strength, balance, and trunk-pelvic dissociation. CG (n = 21): did not receive intervention.	Results: GMFM-66 and GMFM-88 scores improved in both groups (*p* < 0.05). However, EG compared to CG had greater improvement in dimension E and GMFM-66 total score (*p* < 0.05). GMFM-88 scores improved in all dimensions in the EG, but only in dimension B in the CG (*p* < 0.05). The PEDI-FSS total score and its 3 domain scores improved in the EG but not in the CG (*p* < 0.05). Conclusions: hippotherapy produces benefits in gross motor function and functional performance in children with CP compared to CG.
Kwon et al., 2015 [20]	Gross motor function: GMFM-66 and GMFM-88. Balance: PBS	EG (n = 45): received hippotherapy (2 sessions of 30 min × week/8 weeks) as well as conventional physiotherapy. A protocol was used that included muscle relaxation; optimal postural alignment of the head, trunk, and lower extremities; independent sitting; and active exercises (stretching, strengthening, dynamic balance and postural control). CG (n = 46): received aerobic exercise at home, such as walking or cycling, along with conventional physiotherapy (2 sessions of 30 min × week/8 weeks).	Results: The EG, in relation to the CG, showed significant improvements in total GMFM-88, GMFM-66, and GMFM dimensions B, C, D, and E (*p* < 0.05). The GMFM-88 dimensions improved significantly after hippotherapy according to the GMFCS level: dimension E in level I, dimensions D and E in level II, dimensions C and D in level III, and dimensions B and C in level IV. In addition, balance in EG showed improvements in PBS (*p* < 0.05), while no differences were observed in CG. Conclusions: hippotherapy has a positive effect on gross motor skills and balance in children with CP of different functional levels.
Mutoh et al., 2019 [18]	Physical function (Gait parameters): 5-m walk test Gross motor function: GMFM-66 Quality of life (QOL) of patients’ caregivers: WHOQOL-BREF	EG (n = 12): received a hippotherapy program (1 session of 30 min × week/48 weeks) that included muscle relaxation and maintaining optimal postural alignment of the head, trunk, and lower extremities with independent active and seated exercises (stretching, strengthening, dynamic balance and postural control). A 3-month follow-up was performed at the end. CG (n = 12): received a weekly recreational program (1 session of 30 min × week/48 weeks) with leisure activities and children’s games. A 3-month follow-up was conducted at the end of the program. *All children continued their daily routines for the rest of the week, but none received physical therapy during the study.*	Results: In addition to better GMFM-66 (*p* = 0.027) and GMFM-E (*p* = 0.044) scores, hippotherapy was associated with increased cadence, stride length, and mean acceleration (*p* < 0.001); better stabilized horizontal/vertical displacement (*p* = 0.009); and a better relationship between caregiver psychological state and quality of life (*p* < 0.05) compared to CG. In the EG, the improved step length of the children and the psychological quality of life domain of their caregivers were maintained for up to 3 months of follow-up (*p* < 0.05) compared to the CG. Conclusions: A 1-year program of once-weekly hippotherapy can improve the walking ability of children with CP and the psychological health and quality of life of their caregivers, compared to usual daycare recreational activities.
Chinniah et al., 2020 [15]	Motor function in sitting Positions: GMFM-88 dimension B. Measurements were taken every 4, 8, and 12 weeks.	EG (n = 15): received conventional therapy (30 min) and hippotherapy with the simulator (15 min). In the session (3 sessions × week/12 weeks), the children were placed in the saddle and asked to maintain a sitting posture. Mechanical riding therapy produced saddle movements at 3 levels: basic conditioning (flat), forward and backward tilting, and lateral tilting. CG (n = 15): received only conventional physiotherapy based on positioning, stretching, and sitting balance activities (30 min).	Results: GMFM improved in both groups over a period of 12 weeks. Sitting motor function improved gradually over time in both groups. The EG showed a greater improvement in relation to the CG at all weeks (*p* < 0.01). Conclusions: an improvement in sitting motor function was observed in both groups, although the children exposed to the simulator showed a greater improvement. The riding simulator was effective in improving sitting motor function in children with diplegia, and its administration for a longer duration provided more benefit than when used for a shorter duration.
Matusiak-Wieczorek et al., 2020 [17]	Posture and function of each part of the body: SAS	EG 1 (n = 15): 2 sessions of 30 min · week/12 weeks. EG 2 (n = 15): 1 session of 30 min · week/12 weeks. *Program in EG 1 and EG 2: tasks when the horse is standing and then in motion, based on leaning forward and touching the horse’s right ear with the left hand (and* vice versa)*, lifting the straight upper limbs forward, moving sideways and turning the trunk to the right and left, and placing the hands on the back of the head (with elbows apart) to maintain this position throughout the turn.* CG (n = 15): did not receive intervention.	Results: improvements were seen in almost all categories evaluated in the children who participated in hippotherapy. In EG 1, statistically significant differences were observed in the assessment of head position control (*p* = 0.012), arm function (*p* = 0.012), and trunk control (*p* = 0.005), while in EG 2, improvements were observed in the assessment of trunk control (*p* = 0.028). Conclusions: hippotherapy has a positive effect on the posture and function of individual body parts in the seated position in children with CP.
Vidal et al., 2021 [19]	Gross motor function: GMFM–66 Functional performance: PEDI	Group 1 (n = 9): 1 session of 30–35 min × week/16 weeks. Group 2 (n = 11): 2 sessions of 30–35 min × week/16 weeks. *Group 1 and 2: Children wore a protective helmet, sat on the horse, and performed activities to improve forward and upward movement to stimulate postural control, head, trunk, and pelvic movements, strength, and coordination. Activities were performed in sand and outdoor arenas, on tarmac and grass, on different slopes, and in different postures such as classical, lateral, inverted, or quadruped.*	Results: A significant effect was observed in the measures for both groups before and after the evaluation (*p* < 0.05), although no significant differences were reflected when comparing the two groups (*p* > 0.05). Hippotherapy improved gross motor skills and functional performance in children with CP regardless of the frequency of weekly sessions. Conclusions: Significant benefits of hippotherapy were demonstrated in GMFM-66 and PEDI in children with CP; however, a greater effect was observed with twice-weekly treatment.

## Data Availability

Data are contained within the article.
